# Medication Adherence Patterns after Hospitalization for Coronary Heart Disease. A Population-Based Study Using Electronic Records and Group-Based Trajectory Models

**DOI:** 10.1371/journal.pone.0161381

**Published:** 2016-08-23

**Authors:** Julián Librero, Gabriel Sanfélix-Gimeno, Salvador Peiró

**Affiliations:** 1 Health Services Research Unit, Center for Public Health Research (CSISP-FISABIO), Valencia, Spain; 2 Red de Investigación en Servicios de Salud en Enfermedades Crónicas (REDISSEC), Valencia, Spain; Department of Cardiology and Angiology, GERMANY

## Abstract

**Objective:**

To identify adherence patterns over time and their predictors for evidence-based medications used after hospitalization for coronary heart disease (CHD).

**Patients and Methods:**

We built a population-based retrospective cohort of all patients discharged after hospitalization for CHD from public hospitals in the Valencia region (Spain) during 2008 (n = 7462). From this initial cohort, we created 4 subcohorts with at least one prescription (filled or not) from each therapeutic group (antiplatelet, beta-blockers, ACEI/ARB, statins) within the first 3 months after discharge. Monthly adherence was defined as having ≥24 days covered out of 30, leading to a repeated binary outcome measure. We assessed the membership to trajectory groups of adherence using group-based trajectory models. We also analyzed predictors of the different adherence patterns using multinomial logistic regression.

**Results:**

We identified a maximum of 5 different adherence patterns: 1) Nearly-always adherent patients; 2) An early gap in adherence with a later recovery; 3) Brief gaps in medication use or occasional users; 4) A slow decline in adherence; and 5) A fast decline. These patterns represented variable proportions of patients, the descending trajectories being more frequent for the beta-blocker and ACEI/ARB cohorts (16% and 17%, respectively) than the antiplatelet and statin cohorts (10% and 8%, respectively). Predictors of poor or intermediate adherence patterns were having a main diagnosis of unstable angina or other forms of CHD vs. AMI in the index hospitalization, being born outside Spain, requiring copayment or being older.

**Conclusion:**

Distinct adherence patterns over time and their predictors were identified. This may be a useful approach for targeting improvement interventions in patients with poor adherence patterns.

## Introduction

Nonadherence to essential medications has been widely described as a limitation on the effectiveness of evidence-based therapies [1], and has been associated with adverse clinical outcomes and increased health care costs [2–4]. Nonadherence to appropriate medications is a key public health problem of high prevalence, and important gaps in knowledge remain regarding its course and causes and the best ways to improve it.

Traditionally, electronic databases have been used to assess the rates of non-adherence, its predictors and its consequences. Information available in these databases (hospital discharge datasets, electronic medical records, physician order entry systems, electronic prescribing systems, pharmacy claims and others), appropriately combined, enables the construction of observational cohorts for measuring adherence and persistence, and assessment of their impact on clinical outcomes [5,6]. This type of studies usually share three important limitations: 1) they are based on limited groups—such as those affiliated to a particular insurance company or a pharmaceutical benefit scheme—which are not necessarily representative of the general patient population, 2) they use pharmacy claims data with no information about physician prescription and, therefore, they may misclassify patients as non-treated while they actually had a non-filled prescription and, 3) they classify patients into groups of adherence using single indicators (e.g. proportion of days covered [PDC] ≥80%), overlooking the dynamic nature of nonadherence over time.

Advanced electronic prescription systems may provide an opportunity to overcome some of these limitations. The Valencia Health Agency (VHA), the public service responsible for health care in the Autonomous Community of Valencia (Spain), works under a scheme of universal coverage and tax-based funding. The VHA operates an extensive network of hospitals and primary healthcare centers that use a common electronic medical record with an advanced electronic prescription system that, among other features, includes the traceability of the prescription (from physician prescriptions to patients refills at the pharmacy) and provides population-based information.

Methodological alternatives may also add some insights into the study of medication nonadherence. Group-based trajectory models (GBTM) are a type of latent class analysis providing an alternative method for summarizing adherence by incorporating information on its dynamic nature [7]. GBTM is a person-centered approach [8] (as cluster analysis) focused on the relationships among individuals. The most important outputs of the GBTM are the classification of patients into different trajectories over time, and the description of such trajectories over time through easily interpretable graphics.

Despite the extensive use of GBTM in some areas of medical and sociological research [8,9], and its potential for classifying patients according to their long-term adherence [7], it has hardly been used in studies of medication adherence [7,10–16]. Coronary heart disease (CHD) provides an opportunity to explore the potential of these alternatives. In spite of great efforts from the scientific community and professional societies through clear guideline recommendations and quality of care policies based on solid evidence, underprescribing and low adherence to evidence-based therapies remain a significant problem in the management of patients who have suffered hospitalization for CHD [4,17], either because physicians fail to prescribe these medications and/or because patients fail to obtain and/or consume them.

The purpose of his study is to identify adherence patterns over time to four evidence-based medications in all patients discharged alive after hospitalization for CHD using large electronic VHA databases. Additionally, we aim to identify predictors of belonging to the respective adherence patterns.

## Methods

### Design

We constructed a population-based retrospective cohort of all patients discharged alive after an emergency admission for CHD to any VHA hospital in 2008. Patients were followed throughout the health information systems for 9 months after hospital discharge to assess their adherence patterns over time to four therapeutic groups: antiplatelet agents, beta-blockers, drugs acting on the renin-angiotensin system (angiotensin-converting enzyme inhibitors-ACEI- or angiotensin receptor blockers-ARB), and statins.

### Setting

The study was conducted in the Valencia Community, an autonomous region in Spain with 5 million inhabitants in 2008. The Valencia Health Agency covers about 97% of the regional population. During the study period, medical care was free of charge with coverage extending to substantial pharmaceutical benefits: all medicines prescribed to pensioners (eligible because of either age or disability) and underprivileged groups were free of charge. The remaining population paid 40% of the cost of medication (i.e. antiplatelet agents, statins, ACEI or ARB in fixed-dose combinations), but only 10% of the cost with a ceiling of €2.45 (≈$3 USD) per package for some long-term treatments (i.e. beta-blockers, ACEI or ARB).

### Population

The selection and characteristics of the general cohort have been fully described elsewhere [18]. In brief, all patients of both sexes aged 35 years and over, admitted through the Emergency Department and discharged in any of the 24 VHA hospitals with a main diagnosis of acute coronary syndrome (International Classification of Diseases 9th revision Clinical Modification, ICD9CM = 410.xx and 411.xx) or other forms of coronary heart disease (413.xx and 414.xx) between 1 January and 31 December 2008 were included. We excluded deaths in the 30 days following hospital discharge (n = 111), duplicate cases (if the patient had more than one CHD admission, only the first one was accounted for), some government employees whose prescriptions are reimbursed by civil service insurance mutualities not included in the pharmacy databases of the VHA (n = 65), and patients not registered in the municipal census, who left the region or who were discontinued from VHA coverage for other causes (n = 231), because of limitations on follow-up. We also excluded patients who had no contact (no visits, prescriptions, or any other contact) with the VHA within the 9 months after hospital discharge (n = 635) because we suspect that most of those patients could be temporary residents with limitations on follow-up. From the resulting cohort of patients (n = 7462) we created 4 subcohorts with at least one prescription written (regardless of whether the patient filled the prescription or not) from each of the four therapeutic groups within the first 3 months after discharge.

### Data Sources

The main source of data was the VHA ambulatory electronic medical record for ambulatory care, the so-called ABUCASIS system, which among other information includes demographic and clinical data (e.g. diagnoses, personal and family history, lifestyle habits, lab tests/results, etc.), and information on physician prescriptions (what the doctor prescribed) and dispensations from pharmacy claims (what the patient fills from the pharmacy). The information on hospitalizations was based on the Minimum Basic Dataset (MBDS) at hospital discharge, a synopsis of clinical and administrative information on all hospital discharges, including diagnoses and procedures (all electronic health systems in the VHA use the ICD9CM). Mortality, VHA coverage (including dates and causes of VHA discharge) and some demographic characteristics (e.g. age, gender, country of origin, copayment status, etc.) were obtained from the Population Information System.

### Main endpoint

The main outcome measure was monthly adherence to four therapeutic groups (antiplatelet agents, including acetylsalicylic acid (ASA) at doses of 100 mg and clopidogrel, beta-blockers, ACEI/ARB, and statins) based on pharmacy claims (dispensations) according to an ascertainment period of 9 months after hospitalization for CHD. The index date was defined as the date of discharge. We evaluated adherence by creating a “supply diary” for the 270 days (9 months) after the index date. The supply diary was calculated by linking all pharmacy claims based on the dispensing date and the days’ supply [7]. The days’ supply was estimated using the dosing schedule specified in the prescription (one tablet every 8, 12 or 24 hours) and the number of pills per package/prescribed. When this did not agree with standard dosing (around 1% of cases) we rounded up/down the dosing prescribed to the closest frequency (8, 12 or 24 h). Overall, in the Valencia Health System, most of the prescriptions provide 30 days’ supply (around 97.5% of prescriptions provide 30 days supply or less, and the rest provide 60 days supply). If a dispensing occurs before the previous dispensing should have run out, we assume that the new dispensing begins the day after the end of the old dispensing, and days with a drug supply are accumulated. Monthly adherence was defined as having ≥24 days covered out of 30 (≥80% covered), leading to a repeated binary outcome measure at 9 time periods for each drug group and patient, there being 512 theoretically possible adherence patterns. In case of dual antiplatelet therapy, we used a non-restrictive approach, considering patients as adherent to antiplatelet agents if they were adherent to at least one of them (ASA or clopidogrel). Hospitalization days were not taken into account to estimate adherence, as they have been shown to have little impact on adherence to these medications in patients after a myocardial infarction, and are expected to have lower impact in short follow-up periods. [19]

### Covariates

We searched socio-demographic and clinical data (inpatient and outpatient) from the various electronic datasets. We identified the following variables at the time of discharge: age at hospital admission, main admission diagnosis (acute myocardial infarction, angina pectoris, other acute coronary heart disease and other chronic coronary heart disease), gender, country of birth (coded as Spain or other), copayment status, and presence of chronic conditions (hypertension, hyperlipidemia, diabetes, smoking, arrhythmias, congestive heart failure, chronic obstructive pulmonary disease (COPD), chronic renal failure, peripheral vascular disease, cerebrovascular disease, dementia and cancer).

### Ethics

The study was approved by the Ethics and Clinical Trials Committee of the Public Health General Directorate and the Center for Public Health Research. All patient data were transferred to the research team anonymized and de-identified prior to analysis. The Regulatory Commission of Access to Ambulatory Care Information Access of the Valencia Health Authority approved the cession of anonymised data.

### Analysis

First, socio-demographic and clinical characteristics are shown as percentages for the four therapeutic subcohorts. Second, adherence patterns to essential medications after hospitalization for CHD over time were identified using group-based trajectory modeling. Monthly trajectories were modeled with quadratic polynomial functions of time. Each subpopulation is characterized by an intercept, a lineal and quadratic slope allowing for curved developmental patterns. Variance and covariances of these parameters are fixed at zero. The heterogeneity (i.e. the distribution of individual differences within the data) is summarized by a finite set of unique polynomial functions, each corresponding to a discrete trajectory [20].

In the modeling procedure, seven trajectory models were estimated and compared in a stepwise procedure. The selection of the number of trajectory groups or latent classes that best represents the heterogeneity in adherence was based on: 1) model fit indices: Bayesian information criteria (BIC) [21], Akaike information criterion (AIC) [22] where a lower index value indicates a better model fit, and the Lo-Mendell-Rubin likelihood ratio test (LMR-LRT) [23], which tests the improvement in fit between neighboring class models (comparing k-1 and the k class models). LMR-LRT test provides a p-value that can be used to determine if there is a statistically improvement in fit for the inclusion of one more class. A p-value >0.05 was used to reject a new class model; 2) a minimum proportion of the study sample in a class: 5%; and 3) entropy, which indicates uncertainty in the classification of the model and is a measure of how well—or how precisely—study participants are classified into their most likely class. The cut-off value used was a probability of ≥0.7. In summary, the criteria for rejecting k class models (and, thus, selecting k-1 class models) was the presence of some of the following criteria: BIC or AIC score higher; LMR-LRT p-value>0.5; entropy (minimum membership probability) <0.7; and minimum sample size <5%. Once the final number of latent classes was decided, individuals were classified into their most likely class (i.e. the one with the highest membership probability) resulting in an observed (categorical) variable denoting class membership. Patients who died were censored at the date of death; from that date on, they had missing values, contributing to the trajectories and being classified into the different groups based on their adherence behavior during the period they were alive. As a sensitivity analysis, the 9-month PDC (as continuous and binary measure) has been described for each trajectory or latent class in each of the therapeutic subcohorts.

Additionally, we assessed trajectories of adherence to three or more index medications after hospitalization for CHD over time using group-based trajectory modeling as described above. Also, a multiple correspondence analysis (MCA) was used to explore the relationship between the adherence trajectories of the different medications. MCA allows the detection and graphical representation of the underlying structure (dimensions) in a given data set. Interpretation of the results provided by MCA is done based on the graphics; the relative position of the category points indicates the level of association between the categories. The closer the points are, stronger is the relationship between the categories.

Finally, we studied, for each therapeutic group, possible predictors of latent class membership (i.e. factors that characterize the adherence trajectories from each other) using multinomial logistic regression analyses. Their coefficients are the increase in the log-odds of being in a particular trajectory group, versus the nearly-always adherence group, for a one-unit increase in the predictor variable. These were transformed to odds ratios (OR) and their respective 95% confidence intervals. All the analyses were made using Mplus version 6.12 [24] and R [25].

## Results

From the initial cohort of 7462 patients discharged after hospitalization for CHD (median follow-up: 9 months; range, 1–9 months), with at least one primary care physician’s visit and at least one prescription from any of the four therapeutic groups in the complete study period, we generated the 4 subcohorts of patients with at least one prescription written (regardless of whether the patient filled the prescription or not) of the respective medication during the first 3 months after discharge: antiplatelet agents (n = 6513; 87.3% of the initial cohort), beta-blockers (n = 5020; 67.3%), angiotensin antagonists (n = 5243; 70.3%) and statins (n = 6029; 80.8%). Overall, 76.6% of patients received a prescription of three or more drugs of the four therapeutic groups in the first 3 months after hospitalization for CHD. The main characteristics of each cohort are described in Table 1.

**Table 1:** Patient characteristics of the four medication cohorts^a^.

		Antiplatelet cohort (n = 6513)	Beta-blocker cohort (n = 5020)	ACEI/ARB cohort (n = 5243)	Statin cohort (n = 6029)
Age	<45 years	4.0	4.1	3.2	4.0
	45 to 64	33.5	35.5	31.1	34.2
	65 to 79	40.9	41.2	42.4	41.4
	80 and over	21.7	19.2	23.4	20.4
Gender^a^	Male	70.5	70.1	67.5	70.2
	Female	29.5	29.9	32.5	29.8
Country of birth	Spain	89.3	88.5	89.5	89.1
	Other	10.7	11.5	10.5	11.0
Copayment	Yes	25.7	27.2	23.1	26.2
	No	74.3	72.8	76.9	73.8
Main diagnosis at discharge	AMI	52.7	52.5	52.6	52.2
	Unstable angina	15.2	14.5	14.8	15.2
	Stable angina	13.1	13.5	14.2	13.4
	Other CHD	19.0	19.5	18.4	19.2
Comorbidities	Hypertension	62.4	63.6	70.7	62.9
	Hyperlipidemia	42.8	44.0	42.7	45.6
	Diabetes	34.6	35.1	38.4	35.3
	Smoking	25.6	25.3	22.5	25.6
	Arrhythmias	19.3	18.7	21.6	19.6
	Heart failure	13.9	14.1	16.0	13.7
	COPD	7.3	4.3	7.1	6.8
	Chronic renal failure	4.7	4.5	4.8	4.8
	Peripheral vascular dis.	3.5	3.2	3.5	3.5
	Stroke	2.5	2.4	2.6	2.5
	Dementia	0.9	0.6	1.0	0.7
	Cancer	0.9	0.9	0.8	0.8

AMI, acute myocardial infarction; COPD, chronic obstructive pulmonary disease; CHD, coronary heart disease; ACEI, angiotensin-converting enzyme inhibitors; ARB, angiotensin receptor blockers.

^a^All values are expressed as percentages.

Overall, as expected, the cohorts are very similar to each other except for the ACEI/ARB cohort, where patients are more likely to be older, to be females and to be sicker. Patients in the beta-blocker cohort were less likely to have COPD.

**Fig 1:**
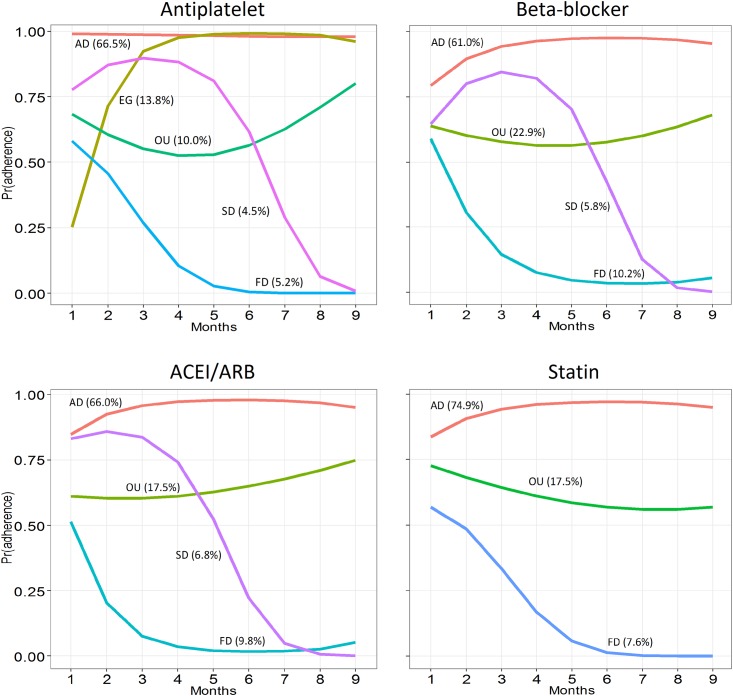
Adherence trajectory patterns for the four cohorts. AD: adherent; EG: early gap; OU: occasional users; SD: slow decline; FD: fast decline, ACEI: angiotensin-converting enzyme inhibitors; ARB: angiotensin receptor blockers.

Combining formal statistical model fit indices (AIC, BIC, Lo-Mendell-Rubin likelihood ratio test) and usefulness criteria (substantive interpretation, minimum size and discriminative properties—entropy) described in Table A in S1 File, we identified five differential adherence trajectories for the antiplatelet cohort, four for the beta-blocker and ACEI/ARB cohorts, and three for the statin cohort (Fig 1). These patterns can be described as follows: 1) Nearly-always adherent patients during the 9-month follow-up period, present in all cohorts; 2) Early gap in adherence after discharge with later recovery, present only in the antiplatelet cohort; 3) Brief gaps in medication use or occasional users, present in all cohorts; 4) Slow decline adherence, present in all cohorts except in the statin cohort; and 5) Fast decline, present in all cohorts.

These patterns characterize variable proportions of patients depending on the therapeutic group (Fig 1; Table 2), the descending trajectories being more frequent for the beta-blocker and ACEI/ARB cohorts (16% and 17%, respectively) compared with the antiplatelet and statin cohorts (10% and 8%, respectively).

**Table 2:** Adherence for the 9-month follow-up for each trajectory and therapeutic group.

		Percentage	mean PDC	PDC>75%
**Antiplatelet**	Adherent	66.5	96.1	97.6
	Early gap	13.8	85.8	84.7
	Occasional users	10.0	60.9	28.0
	Slow decline	4.5	54.6	9.2
	Fast decline	5.2	16.4	0.0
**Beta-blockers**	Adherent	61.0	94.1	96.1
	Occasional users	22.9	61.0	20.6
	Slow decline	5.8	52.4	8.6
	Fast decline	10.2	19.3	0.6
**ACEI/ARB**	Adherent	66.0	92.4	91.4
	Occasional users	17.5	61.4	20.7
	Slow decline	6.8	45.4	2.5
	Fast decline	9.8	11.9	0.2
**Statins**	Adherent	74.9	92.9	93.6
	Occasional users	17.5	58.9	14.5
	Fast decline	7.6	17.5	0.0

PDC, percentage of days covered

The nearly-always adherent trajectory was more frequent in the statin cohort (74.9% of patients) than in other cohorts (61.0% to 66.5% of patients). However, for the antiplatelet cohort, an additional 13.8% of patients have good adherence overall despite a short early gap.

On the other hand, the probability of being fully adherent in the first-month of follow-up is quite consistent among analogous or similar trajectories in the four therapeutic groups (Fig 1): 80–100% for the nearly-always adherent trajectories, 60–75% for the occasional users, 65–80% for the slow decline and 50–60% for the fast decline trajectories. For the early gap trajectory, only present for antiplatelet agents, this probability was only 25%.

The 9-month PDC (as continuous and binary measure) for each trajectory or latent class in each of the therapeutic subcohorts is displayed in Table 2, showing high consistency between the adherence trajectories identified and the 9-month PDC.

The relationship between the adherence trajectories of the different medications is shown in a multiple correspondence analysis plot (Figure A in S1 File). Additionally, trajectory models considering a composite outcome of adherence to 3 or more of the 4 index medications assessed are shown in Figure B in S1 File.

**Fig 2:**
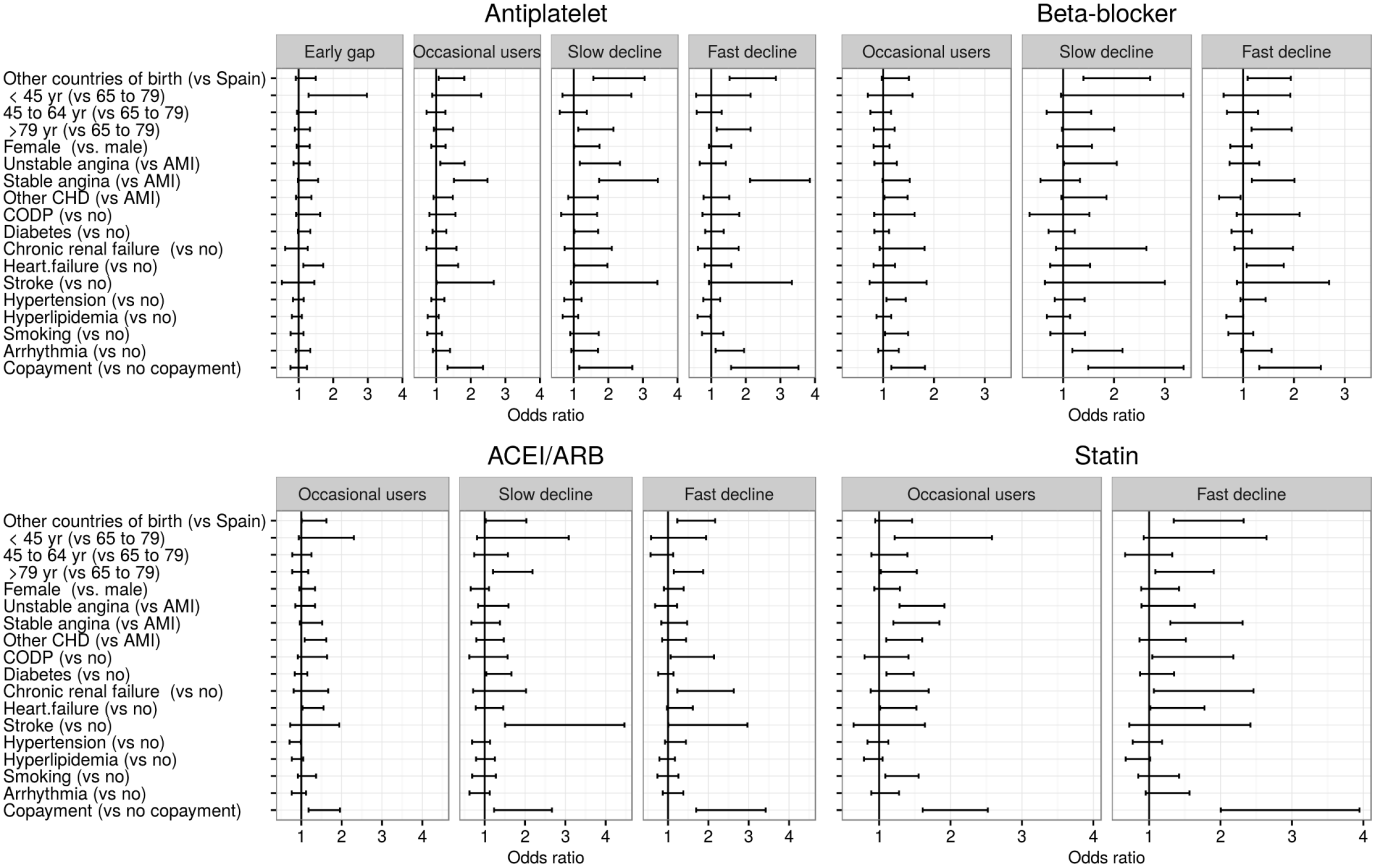
Predictors of poor or intermediate adherence trajectory groups. Multinomial logistic regression analysis. ACEI: angiotensin-converting enzyme inhibitors; ARB: angiotensin receptor blockers; CHD: coronary heart disease; AMI: acute myocardial infarction; COPD: chronic obstructive pulmonary disease. The reference category is the nearly-always adherent trajectory group. Estimates for peripheral vascular disease, cancer and dementia were not included due to their high random error.

Regarding potential predictors of being included in one or another adherence trajectory (Fig 2; Tables B-E in S1 File), patients belonging to the occasional users pattern, when compared with nearly-always adherent patients, were more likely to have a main diagnosis of unstable angina or other forms of CHD in the index hospitalization and to have copayment in all cohorts, and patients in the slow or fast declining trajectories (a slow decline pattern was not present in the statin cohort) were, in general, more likely to be born outside Spain, to be over 79 years old and to have copayment. In the early gap pattern, only present in the antiplatelet cohort, patients were more likely to be younger compared with nearly-always adherent patients (OR<45yr: 1.96).

## Discussion

In the present study, through group-based trajectory models, we identified distinct adherence patterns to secondary prevention medications over time in a population-based cohort of patients after hospitalization for CHD. This approach showed interesting advantages over traditional methods of adherence assessment, which often overlook the dynamic nature of medication adherence. Moreover, we were able to identify some characteristics associated with poor or intermediate adherence patterns, which could enable clinicians and researchers to target improvement interventions more accurately.

First at all, this study shows that between one third (for antiplatelet, beta-blockers and ACEI/ARB) and one quarter (for statins) of patients after hospitalization for CHD, who were prescribed the corresponding drug by their physicians in the immediate post-discharge period, exhibit some form of adherence gap in the nine following months. To the best of our knowledge, there are no studies assessing medication adherence after hospitalization for CHD using GBTM. Traditional studies assessing adherence to secondary prevention pharmacotherapy after an ACS showed suboptimal figures (ranging from 54% to 86% at one year) [17], although such figures are not directly comparable to those of the present study, as they do not provide information on adherence behaviors over time.

Group-based trajectory models enabled the identification of five differential patterns, which varied depending on the therapeutic group assessed. Among patterns common to all therapeutic classes, we found a prevailing group of nearly-always adherent patients; a group with brief gaps in adherence accounting for 10–20% of patients depending on the therapeutic class; and a small non-adherent group with a fast decline in medication use representing 5–10% of cases. Regarding patterns specific to particular therapeutic classes, we found a group of patients showing a slow decline adherence over time (4–7% of patients, not present in the statin cohort), and a relevant group (14% of patients) with a short early gap and later recovery in adherence, only identified in the antiplatelet cohort.

It is of interest to note that although the extreme adherence groups would probably have been easy to identify by conventional methods of adherence assessment, such as PDC, the intermediate patterns of adherence (occasional users, slow decline adherence, and early gap and later recovery) would have been rated very similarly by these methods, which establish averages for the entire follow-up period rather than considering the temporal dynamics of adherence. They would thus group patients with very different adherence behavior over time into the same category. Likewise, the usual methods for persistence assessment (time to the first gap of n days, days of uninterrupted therapy) would have incorrectly classified groups such as “early gap with later recovery” or “occasional users”. In this sense, the use of group-based trajectory models seems to produce comprehensive and easily understandable outputs that would require the combined handling of several conventional single indicators. It is precisely in these areas that adherence modelization by GBTM methods has noticeable advantages compared with traditional methods of adherence assessment.

The genuine practical usefulness of these methods, however, should be determined by their ability to identify patients with a high probability of following certain adherence trajectories, enabling the implementation of more effective adherence improvement interventions. Unfortunately, the multinomial models identified few predictors of poor adherence trajectories. This was an expected result given that administrative databases do not usually provide data regarding some factors thought to determine adherence, such as educational level or the interaction between patients and physicians, making it difficult to predict adherence [26,27]. Nevertheless, we were able to identify some relevant predictors of poor or intermediate adherence patterns, such as being born outside Spain, having copayment or being older. This is an interesting finding because recently, and as part of the measures taken to reduce the public deficit, the Spanish Government has introduced widespread copayments and restrictions on non-emergency care to immigrants, which could contribute to increasing nonadherence in disadvantaged populations. It is a particularly worrying situation in conditions like secondary CHD prevention where a decrease in adherence can have a high impact on health (and on the costs associated with hospital readmissions) [4, 28], probably requiring an accurate assessment of their potential impact on population health and health expenditure.

Overall, this study suggests that poorly adherent groups have different patterns and would probably benefit from very different interventions. Patients having problems at the beginning of the therapy (engaging to the therapy) would probably benefit from interventions focused on better explanation of the importance of disease and/or taking the medication appropriately, lower copayments, etc.; while patients having problems later on (e.g. slow decline) may benefit from interventions focused on reminders and similar. The characteristics identified may facilitate the targeting of such interventions in specific subgroups.

### Limitations

Some potential limitations of this study should be addressed. Regarding the study design, the possible sources of bias include: 1) our study has evaluated adherence in patients with at least one prescription in the 90 days after hospital discharge, thus not taking into account that for some patients the doctor could have suspended the prescription afterwards, due to side effects or for other reasons; 2) we used pharmacy claims for measuring adherence, but patients do not necessarily consume all the drugs they fill. Nevertheless, several studies have shown a high consistency between dispensation and patient consumption [29,30]; 3) ASA is an over-the-counter and low cost drug that could be acquired without prescription and thus could not be reliably captured in pharmacy claims; 4) dispensations obtained from pharmacies outside the Valencia region (e.g. on holidays outside the region) or during hospitalization (drugs are provided at no cost by the Hospital Pharmacy Service) have not been recorded in our study. 5) Refills previous to the index admission and hospitalization days after the index event were not taken into account, however, the latter have been shown that have little impact on adherence in post-MI patients to these medications, even in short follow-up periods [19]. Some of these design problems tend to overestimate nonadherence while others tend to underestimate it, but overall no major impact is expected on the identification of adherence patterns.

Regarding the analysis, some limitations should be mentioned with respect to latent class models [31–34]: 1) the modeling process includes several choices and each choice influences the final number of classes and/or their characteristics. For example, if the within-class variance parameters are fixed at zero, as in GBTM, then the final number of classes will often be larger than in models allowing freely estimated within-class variance parameters. In the same way, several features of the study design, such as sample size and the number of measurements, could also influence the number and characteristics of classes identified in the final model [23,35,36]. Moreover, the inconsistency of the model fit indices adds extra challenges in determining the final number of classes. Although simulation studies [37] suggest the relative advantage of some model fit indices, clear guidelines for building an appropriate latent class model are lacking; 2) the identification of distinct classes is no proof of the true existence of multiple subpopulations [20,23,38,39]. As in all statistical models, the final model is just a simplification of a complex reality, an issue that should be kept in mind when conducting latent class models.

Regarding adherence predictors, as stated previously, information from administrative databases and ambulatory electronic medical records do not include some of the variables that could influence adherence (i.e. a patient’s motivation or certain side effects such as fatigue or sexual dysfunction), and other relevant covariates such as procedures during the index event or prior ischemic events were not available. However, we were able to identify some important characteristics related to nonadherence. Finally, regarding the external validity of our findings, the present study was conducted in a specific region. Given the potential differences with other regions or countries, the generalization of our findings to other settings should be made prudently, as should the generalization to other drugs or types of patients.

### Conclusions

We were able to identify, through group-based trajectory models, distinct adherence patterns over time beyond the classic approaches, which often overlook the dynamic nature of adherence. We also identified some predictors of these patterns, which could enable clinicians and researchers to design more appropriate and effective improvement interventions in patients with poor or intermediate adherence patterns.

## Supporting Information

S1 File Online supporting information. Criteria to determine the number of trajectories that best represent adherence patterns (**Table A**); Predictors of poor or intermediate adherence trajectory groups (**Tables B-E**); Multiple correspondence analysis plot for the adherence trajectories of the four therapeutic groups (**Figure A**); Adherence trajectory patterns to three or more therapeutic groups (**Figure B**).
(PDF)
