# Supplementary material for: Medication Adherence Patterns after Hospitalization for Coronary Heart Disease. A Population-Based Study Using Electronic Records and Group-Based Trajectory Models
Source: PLoS One. 2016 Aug 23;11(8):e0161381. doi: 10.1371/journal.pone.0161381 (PMC4995009; doi:10.1371/journal.pone.0161381)

Supporting Information

SUPPLEMENTARY TABLES

Table A. Criteria to determine the number of trajectories that best represent adherence patterns for the different therapeutic groups.

		AIC	BIC	LMR-LRT p-value	Entropy	Minimum size
Antiplatelet	1 trajectory	44674.64	44694.99			
	2 trajectories	34193.27	34240.74	0.00	0.93	0.16
	3 trajectories	33145.46	33220.06	0.00	0.84	0.07
	4 trajectories	32623.67	32725.39	0.00	0.86	0.06
	**5 trajectories**	**32258.50**	**32387.35**	**0.00**	**0.83**	**0.05**
	6 trajectories	32123.02	32279.00	0.15	0.73	0.04
	7 trajectories	32019.33	32202.43	0.76	0.74	0.02
Beta-blockers	1 trajectory	49388.34	49407.90			
	2 trajectories	40104.29	40149.94	0.00	0.88	0.31
	3 trajectories	39054.20	39125.93	0.00	0.85	0.09
	**4 trajectories**	**38763.30**	**38861.12**	**0.00**	**0.83**	**0.06**
	5 trajectories	38697.35	38821.25	0.00	0.69	0.06
	6 trajectories	38441.08	38591.07	0.00	0.69	0.05
	7 trajectories	38305.65	38481.72	0.00	0.69	0.04
ACEI/ARB	1 trajectory	48741.65	48761.35			
	2 trajectories	37552.95	37598.91	0.00	0.91	0.24
	3 trajectories	36223.22	36295.43	0.00	0.84	0.11
	**4 trajectories**	**35611.82**	**35710.29**	**0.00**	**0.83**	**0.07**
	5 trajectories	35380.51	35505.24	0.23	0.85	0.04
	6 trajectories	35200.70	35351.68	0.00	0.81	0.03
	7 trajectories	35043.57	35220.82	0.00	0.74	0.04
Statins	1 trajectory	49039.28	49059.40			
	2 trajectories	39819.18	39866.11	0.00	0.91	0.18
	**3 trajectories**	**39003.50**	**39077.24**	**0.00**	**0.84**	**0.08**
	4 trajectories	38864.56	38965.13	0.00	0.66	0.06
	5 trajectories	38441.97	38569.35	0.00	0.71	0.05
	6 trajectories	38169.77	38323.97	0.16	0.64	0.06
	7 trajectories	38005.69	38186.71	0.00	0.76	0.03

AIC: akaike information criterion; BIC: bayesian information criteria; LMR-LRT: Lo-Mendell-Rubin likelihood ratio test; ACEI: angiotensin-converting enzyme inhibitors; ARB: angiotensin receptor blockers.
In bold the models selected after fulfilling all criteria. The criteria for rejecting k class models (and, thus, selecting k-1 class models) was the presence of some of the following criteria: BIC or AIC score higher; LMR-LRT p-value>0.5; entropy (minimum membership probability) <0.7; and minimum sample size <5%.

Table B. Predictors of poor or intermediate adherence trajectory groups for antiplatelet. Multinomial logistic regression analysis.

		Early gap		Occasional users		Slow decline		Fast decline	
		OR	95%CI	OR	95%CI	OR	95%CI	OR	95%CI
Age	<45 years	**1.96**	**(1.29-2.97)**	1.42	(0.88-2.30)	1.34	(0.67-2.67)	1.06	(0.56-2.14)
	45 to 64	1.19	(0.95-1.49)	0.94	(0.72-1.27)	0.90	(0.60-1.38)	0.83	(0.58-1.30)
	65 to 79	1.00		1.00		1.00		1.00	
	80 and over	1.08	(0.80-1.33)	1.18	(0.93-1.49)	**1.56**	**(1.13-2.15)**	**1.62**	**(1.16-2.14)**
Gender	Male	1.00		1.00		1.00		1.00	
	Female	1.11	(0.94-1.32)	1.05	(0.90-1.29)	**1.34**	**(1.01-1.75)**	1.20	(0.94-1.58)
Country of birth	Spain	1.00		1.00		1.00		1.00	
	Other	1.17	(0.92-1.49)	**1.38**	**(1.07-1.81)**	**2.20**	**(1.57-3.05)**	**2.14**	**(1.53-2.87)**
Copayment	Yes	0.97	(0.76-1.20)	**1.79**	**(1.33-2.35)**	**1.74**	**(1.33-2.35)**	**2.5**	**(1.57-3.52)**
	No	1.00		1.00		1.00		1.00	
Main diagnosis at discharge	AMI	1.00		1.00		1.00		1.00	
	Unstable angina	1.06	(0.85-1.32)	**1.43**	**(1.12-1.82)**	**1.67**	**(1.12-1.82)**	1.01	(0.67-1.40)
	Stable angina	1.23	(0.98-1.56)	**1.94**	**(1.51-2.48)**	**2.47**	**(1.74-3.43)**	**2.96**	**(2.12-3.85)**
	Other CHD	1.12	(0.92-1.37)	1.17	(0.92-1.48)	1.21	(0.92-1.48)	1.09	(0.78-1.52)
Comorbidities	Hypertension	0.98	(0.83-1.15)	1.03	(0.86-1.24)	0.94	(0.73-1.23)	0.97	(0.77-1.26)
	Hyperlipidemia	0.94	(0.80-1.09)	0.90	(0.75-1.07)	0.87	(0.68-1.13)	**0.75**	**(0.61-0.98)**
	Diabetes	1.14	(0.98-1.34)	1.08	(0.90-1.29)	**1.31**	**(1.07-1.81)**	1.05	(0.82-1.36)
	Smoking	0.94	(0.77-1.14)	0.92	(0.74-1.16)	1.27	(0.90-1.72)	1.02	(0.73-1.35)
	Arrhythmias	1.11	(0.92-1.34)	1.13	(0.90-1.40)	1.26	(0.93-1.70)	**1.50**	**(1.13-1.95)**
	Heart failure	**1.39**	**(1.13-1.71)**	1.26	(0.99-1.63)	**1.41**	**(1.01-1.97)**	1.07	(0.81-1.58)
	COPD	1.23	(0.93-1.62)	1.12	(0.80-1.56)	1.03	(0.64-1.68)	1.18	(0.75-1.81)
	Chronic renal failure	0.88	(0.61-1.26)	1.07	(0.72-1.59)	1.25	(0.74-2.10)	1.01	(0.62-1.79)
	Stroke	0.86	(0.51-1.45)	**1.64**	**(1.01-2.60)**	1.78	(0.92-3.42)	1.83	(0.94-3.33)

AMI: acute myocardial infarction; CI: confidence interval; COPD: chronic obstructive pulmonary disease; IHD: ischemic heart disease, pulmonary disease; OR: odds ratio.

The reference category is the nearly-always adherent trajectory group. In bold significant covariates (p<0.05) are marked. Estimates for peripheral vascular disease, cancer and dementia were not included due to their high random error.

Table C. Predictors of poor or intermediate adherence trajectory groups for beta-blocker. Multinomial logistic regression analysis.

		Occasional users		Slow decline		Fast decline	
		OR	95%CI	OR	95%CI	OR	95%CI
Age	<45 years	1.05	(0.70-1.58)	1.79	(0.96-3.36)	1.08	(0.62-1.93)
	45 to 64	0.93	(0.75-1.16)	1.02	(0.67-1.55)	0.92	(0.68-1.29)
	65 to 79	1.00		1.00		1.00	
	80 and over	1.00	(0.82-1.23)	1.40	(0.98-2.00)	**1.51**	**(1.17-1.95)**
Gender	Male	1.00		1.00		1.00	
	Female	0.96	(0.81-1.13)	1.18	(0.89-1.57)	0.94	(0.75-1.17)
Country of birth	Spain	1.00		1.00		1.00	
	Other	1.21	(0.97-1.51)	1.95	(0.86-2.64)	**1.46**	**(1.09-1.94)**
Copayment	Yes	**1.45**	**(1.16-1.82)**	**2.25**	**(1.49-3.37)**	**1.84**	**(1.32-2.53)**
	No	1.00		1.00		1.00	
Main diagnosis at discharge	AMI	1.00		1.00		1.00	
	Unstable angina	1.02	(0.83-1.27)	**1.45**	**(1.02-2.06)**	0.99	(0.73-1.32)
	Stable angina	1.23	(0.99-1.52)	0.86	(0.55-1.33)	**1.55**	**(1.17-2.01)**
	Other CHD	**1.23**	**(1.03-1.48)**	1.34	(0.96-1.85)	**0.72**	**(0.53-0.95)**
Comorbidities	Hypertension	**1.24**	**(1.07-1.45)**	1.09	(0.83-1.42)	1.16	(0.95-1.44)
	Hyperlipidemia	1.00	(0.87-1.16)	0.88	(0.68-1.14)	**0.81**	**(0.67-0.99)**
	Diabetes	0.96	(0.83-1.12)	0.94	(0.71-1.23)	0.94	(0.77-1.17)
	Smoking	**1.24**	**(1.04-1.49)**	1.03	(0.75-1.43)	0.93	(0.71-1.20)
	Arrhythmias	1.09	(0.90-1.31)	**1.60**	**(1.18-2.17)**	1.23	(0.96-1.56)
	Heart failure	1.00	(0.81-1.23)	1.07	(0.74-1.53)	**1.37**	**(1.07-1.80)**
	COPD	1.16	(0.82-1.62)	0.72	(0.34-1.52)	1.36	(0.87-2.11)
	Chronic renal failure	1.30	(0.93-1.82)	1.51	(0.86-2.64)	1.29	(0.83-1.98)
	Stroke	1.17	(0.73-1.86)	1.39	(0.64-3.00)	1.54	(0.88-2.69)

AMI: acute myocardial infarction; CI: confidence interval; COPD: chronic obstructive pulmonary disease; IHD: ischemic heart disease, pulmonary disease; OR: odds ratio.

The reference category is the nearly-always adherent trajectory group. . In bold significant covariates ($p<0.05$) are marked. Estimates for peripheral vascular disease, cancer and dementia were not included due to their high random error.

Table D. Predictors of poor or intermediate adherence trajectory groups for ACEI/ARB. Multinomial logistic regression analysis.

		Occasional users		Slow decline		Fast decline	
		OR	95%CI	OR	95%CI	OR	95%CI
Age	<45 years	1.47	(0.93-2.30)	1.59	(0.81-3.08)	1.05	(0.58-1.94)
	45 to 64	0.98	(0.77-1.25)	1.08	(0.74-1.57)	0.79	(0.57-1.13)
	65 to 79	1.00		1.00		1.00	
	80 and over	0.95	(0.77-1.17)	**1.62**	**(1.21-2.19)**	**1.48**	**(1.14-1.87)**
Gender	Male	1.00		1.00		1.00	
	Female	1.13	(0.95-1.34)	0.85	(0.65-1.11)	1.12	(0.90-1.39)
Country of birth	Spain	1.00		1.00		1.00	
	Other	**1.28**	**(1.01-1.62)**	**1.45**	**(1.03-2.03)**	**1.64**	**(1.23-2.17)**
Copayment	Yes	**1.52**	**(1.18-1.96)**	**1.79**	**(1.23-2.67)**	**2.46**	**(1.70-3.42)**
	No	1.00		1.00		1.00	
Main diagnosis at discharge	AMI	1.00		1.00		1.00	
	Unstable angina	1.07	(0.85-1.34)	1.16	(0.84-1.59)	0.92	(0.68-1.22)
	Stable angina	1.21	(0.98-1.52)	0.97	(0.67-1.38)	1.12	(0.83-1.47)
	Other CHD	1.32	(1.08-1.62)	1.09	(0.79-1.47)	1.11	(0.85-1.45)
Comorbidities	Hypertension	0.84	(0.71-0.99)	0.88	(0.69-1.13)	1.15	(0.93-1.44)
	Hyperlipidemia	0.89	(0.77-1.05)	0.99	(0.79-1.25)	0.95	(0.79-1.17)
	Diabetes	0.98	(0.84-1.15)	**1.31**	**(1.04-1.66)**	0.92	(0.76-1.14)
	Smoking	1.12	(0.91-1.37)	0.95	(0.69-1.28)	0.97	(0.74-1.26)
	Arrhythmias	0.92	(0.76-1.12)	0.84	(0.62-1.12)	1.09	(0.87-1.38)
	Heart failure	**1.26**	(1.18-1.96)	1.07	(0.78-1.46)	1.23	(0.97-1.62)
	COPD	1.22	(0.91-1.64)	0.99	(0.62-1.57)	**1.52**	**(1.06-2.14)**
	Chronic renal failure	1.16	(0.81-1.67)	1.20	(0.71-2.03)	1.82	(1.23-2.63)
	Stroke	1.19	(0.72-1.94)	**2.60**	**(1.51-4.46)**	1.75	(1.01-2.97)

AMI: acute myocardial infarction; CI: confidence interval; COPD: chronic obstructive pulmonary disease; IHD: ischemic heart disease, pulmonary disease; OR: odds ratio; ACEI: angiotensin-converting enzyme inhibitors; ARB: angiotensin receptor blockers.

The reference category is the nearly-always adherent trajectory group. . In bold significant covariates (p<0.05) are marked. Estimates for peripheral vascular disease, cancer and dementia were not included due to their high random error.

Table E. Predictors of poor or intermediate adherence trajectory groups for statins. Multinomial logistic regression analysis.

		Occasional users		Fast decline	
		OR	95%CI	OR	95%CI
Age	<45 years	**1.79**	**(1.22-2.58)**	1.55	(0.92-2.64)
	45 to 64	1.11	(0.89-1.40)	0.92	(0.66-1.32)
	65 to 79	1.00		1.00	
	80 and over	**1.25**	**(1.02-1.53)**	**1.45**	**(1.09-1.91)**
Gender	Male	1.00		1.00	
	Female	1.1	(0.93-1.29)	1.12	(0.89-1.42)
Country of birth	Spain	1.00		1.00	
	Other	1.18	(0.95-1.46)	**1.79**	**(1.34-2.32)**
Copayment	Yes	**2.02**	**(1.61-2.52)**	**2.85**	**(2.00-3.95)**
	No	1.00		1.00	
Main diagnosis at discharge	AMI	1.00		1.00	
	Unstable angina	**1.57**	**(1.29-1.91)**	1.23	(0.89-1.64)
	Stable angina	**1.49**	**(1.20-1.84)**	**1.76**	**(1.30-2.31)**
	Other CHD	**1.33**	**(1.10-1.60)**	1.15	(0.87-1.51)
Comorbidities	Hypertension	0.97	(0.84-1.13)	0.95	(0.77-1.18)
	Hyperlipidemia	0.91	(0.79-1.05)	0.81	(0.67-1.01)
	Diabetes	**1.28**	**(1.10-1.48)**	1.07	(0.87-1.35)
	Smoking	**1.30**	**(1.09-1.55)**	1.11	(0.85-1.42)
	Arrhythmias	1.07	(0.89-1.28)	1.21	(0.95-1.57)
	Heart failure	**1.24**	**(1.01-1.52)**	**1.33**	**(1.02-1.78)**
	COPD	1.51	(0.80-1.41)	**1.06**	**(1.04-2.18)**
	Chronic renal failure	1.63	(0.88-1.69)	**1.22**	**(1.07-2.46)**
	Stroke	1.33	(0.64-1.64)	1.03	(0.72-2.42)

AMI: acute myocardial infarction; CI: confidence interval; COPD: chronic obstructive pulmonary disease; IHD: ischemic heart disease, pulmonary disease; OR: odds ratio.

The reference category is the nearly-always adherent trajectory group. . In bold significant covariates (p<0.05) are marked. Estimates for peripheral vascular disease, cancer and dementia were not included due to their high random error.

SUPPORTING FIGURES

Figure A. Multiple correspondence analysis (MCA) plot for the adherence trajectories of the four therapeutic groups (N=7,462). AD: adherent; EG: early gap; OU: occasional users; SD: slow decline; FD: fast decline, NP: no prescription; ACEI: angiotensin-converting enzyme inhibitors; ARB: angiotensin receptor blockers. The relative position of the categories indicates the level of association between the categories. The closer the categories are, the stronger is the relationship between the categories. Cumulative variance: 22.8 % for the 2 dimensions plotted.

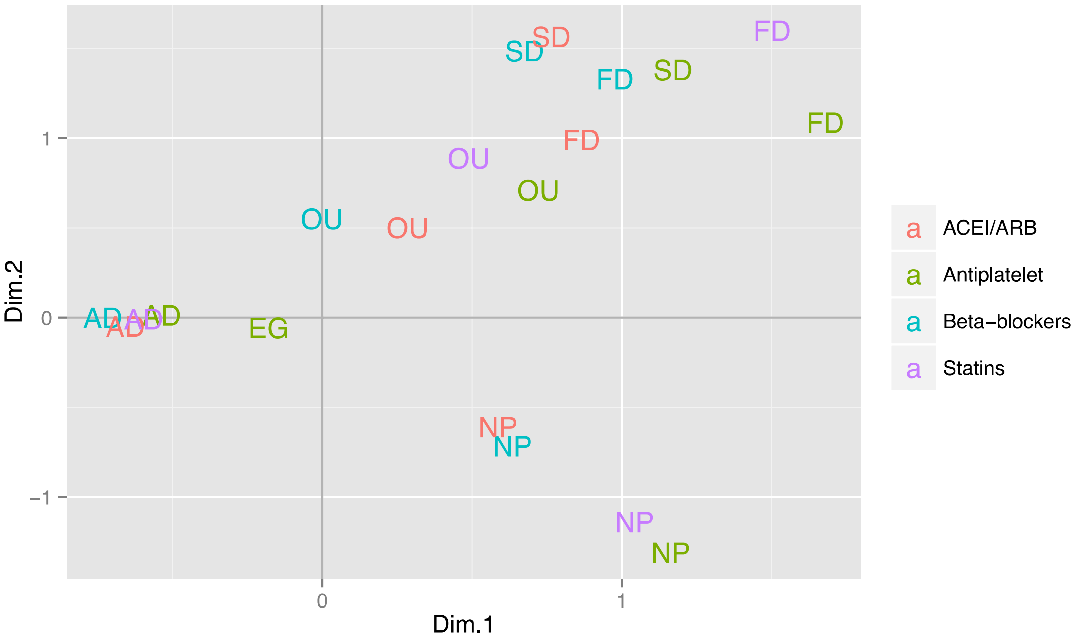

Figure B. Adherence trajectory patterns to three or more therapeutic groups (N= 5,717). AD: adherent; EG: early gap; SD: slow decline; FD: fast decline.

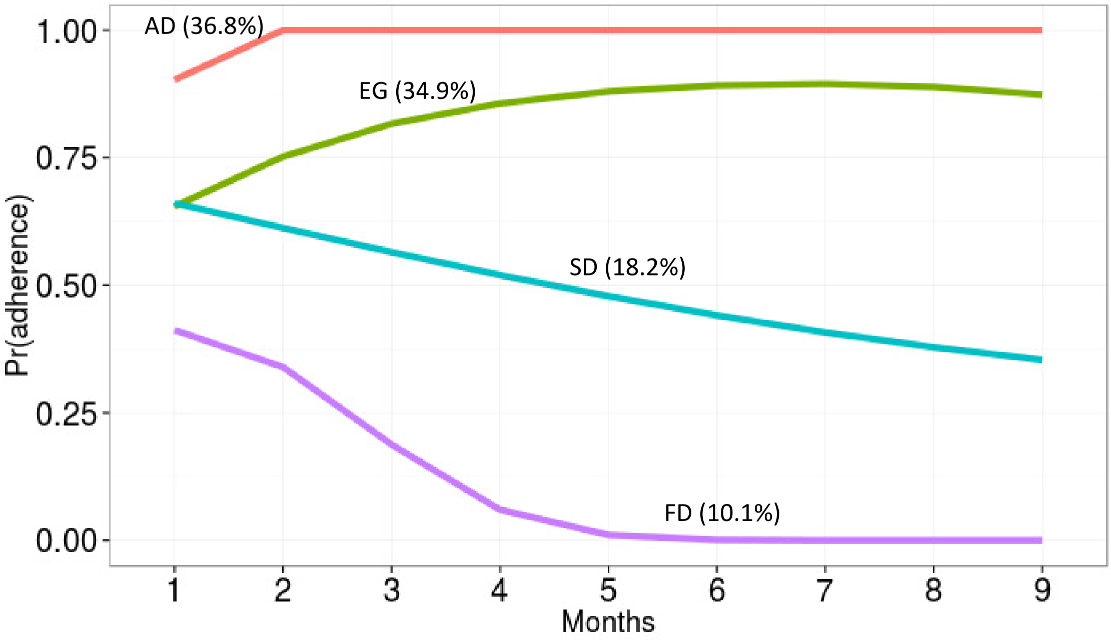

Supplement: S1 File — Criteria to determine the number of trajectories that best represent adherence patterns (Table A); Predictors of poor or intermediate adherence trajectory groups (Tables B-E); Multiple correspondence analysis plot for the adherence trajectories of the four therapeutic groups (Figure A); Adherence trajectory patterns to three or more therapeutic groups (Figure B). (PDF) [file pone.0161381.s001.pdf]
